# Preparation and Characterization of Silk Fibroin Nanoparticles as a Potential Drug Delivery System for 5-Fluorouracil

**DOI:** 10.15171/apb.2019.069

**Published:** 2019-10-24

**Authors:** Hamid Rahmani, Ali Fattahi, Komail Sadrjavadi, Salar Khaledian, Yalda Shokoohinia

**Affiliations:** ^1^Pharmaceutical Sciences Research Center, Health Institute, Kermanshah University of Medical Sciences, Kermanshah, 6734667149, Iran.; ^2^Student Research Committee, School of Pharmacy, Kermanshah University of Medical Sciences, Kermanshah, 6734667149, Iran.; ^3^Medical Biology Research Center, Health Technology Institute, Kermanshah University of Medical Sciences, Kermanshah, Iran.

**Keywords:** SF, 5-Fluorouracile, Nanoparticle, Precipitation, Cytotoxicity

## Abstract

***Purpose:*** The aim of this study is to prepare 5-fluorouracil (5-FU) loaded silk fibroin nanoparticles (SFNPs) and to achieve a controlled release delivery system with the high loading capacity.

***Methods:*** SFNPs with 1:1, 1:3, and 1:10 ratios of 5-FU to silk fibroin were prepared. SFNPs were characterized by Fourier-transform infrared spectroscopy (FT-IR), X-ray diffraction (XRD) analysis, Scanning electron microscope (SEM), and Transmission electron microscope (TEM). Loading efficiency, in vitro release, and cell viability were studied for optimal SFNPs.

***Results:*** The ratio of 1:1 was optimal formulation with the size and polydispersity index (PDI) of 221.03 nm and 0.093 before freeze drying, and 286.7 nm and 0.154 after freeze drying by lactose, respectively. The loading efficiency and loading content of this ratio were 52.32% and 34.35%, respectively. FT-IR and XRD analysis indicated the conformational change (from random coil to β-sheet) in the structure of nanoparticles by increasing amount of the drug, which caused the smaller size, the higher loading efficiency, and the slower release pattern. The drugloaded nanoparticles reached to the half maximal inhibitory concentration (IC50) that were comparable with free drug on MCF7 (human breast cancer) cell line.

***Conclusion:*** This study was planned to achieve a promising controlled release drug delivery system for carrying 5-FU, as a potent anticancer drug. SFNPs were found proper candidates for delivery of a hydrophilic drug such as 5-FU.

## Introduction

Recently, nanoparticles have attracted a myriad of attention in pharmaceutical technology as drug delivery systems. These systems can be used to obtain targeted delivery, enhanced bioavailability, sustained drug release, efficient uptake, etc.^1^ These nano based drug formulations possess the advantages such as reducing the expenses and toxicity, increasing the drug efficiency and tolerability,^2^ and improving the drug solubility.^3-5^

Various polymeric materials can be utilized as a nano based drug delivery system, including the synthetic and natural polymers.^6,7^ The type of material used for nano-carriers plays significant role in loading amount, kinetic of release and destiny of drug in the body. According to advantages of biodegradable polymeric nanoparticles e.g. excellent biocompatibility, valuable encapsulation capacity, and controlled release properties, these materials have been extensively used as the carriers in drug delivery systems.^8,9^

Silk fibroin (SF) is a natural polymer with several unique features which have made it an attractive material for drug delivery. This polymer is biocompatible, biodegradable, and possesses excellent mechanical properties. It is characterized by repetitive sequences of six hydrophobic residues (Gly–Ala–Gly–Ala–Gly–Ser) which form β-sheet and are responsible for the mechanical properties.^10^ The formation of β-sheet leads to insolubility in water and increases the hydrophobicity of the polymer.^11^

5-FU is a uracil nucleotide analog that has been used more than five decades in the treatment of various human malignancies such as stomach, colon, rectum and breast cancers.^12-15^ Due to rapid metabolism in the liver and short half-life of 5-FU, high doses of the drug, e.g. 400-600 mg/m^2^/wk is required to assure the adequate treatment,^16^ but high doses cause toxicity in the gastrointestinal tract and myelotoxicity.^17,18^ As it is resulted from the literature, this drug should be dosed once or twice a week, preferably as a long-acting injection and targeted to the desired site.^19^ Biodegradable polymeric nano-hydrogels could be suitable materials promising the way to achieve this purpose, but their application was limited because of low drug loading and fast drug release, which are attributed to the high water content and large pore size of hydrogels.^20^ The loading efficiency of 5-FU in the most of the nano-hydrogels e.g. chitosan and alginate nanoparticles was lower than 30%, and even if the loading efficiency was higher, it was decreased by increasing the drug to Polymer ratio.^21,22^ Therefore producing a nano-hydrogel system with high capacity for drug loading and sustainability for water soluble small molecules such as 5-FU can introduce a new generation of nanoparticles to the pharmaceutical industries for effective sustained delivery of water soluble drugs by the hydrogels.

The present work essentially focused on designing a drug delivery system using SF and examining the ability of this polymer in encapsulating of 5-FU, to produce a controlled release formulation of this drug with high loading efficiency. Nanoprecipitation as an instantaneous, simple and one step method^23^ was used to obtain SFNPs. The cytotoxic effect of these nanoparticles on MCF7 and HT29 (human colon adenocarcinoma) cell lines was also investigated.

## Materials and Methods

### Materials

Silk cocoons were purchased from local market of Tabriz (Tabriz, Iran). DMSO, mannitol, sorbitol, PEG 4000, and lactose were procured from Merck (Darmstadt, Germany). 5-FU was purchased from Sigma–Aldrich (St. Louis, MO, USA). Sodium carbonate and Lithium bromide (LiBr) were supplied from Scharlau (Sentmenat, Spain) and from Ridel-de Haen (Seelze, Germany), respectively. Acetone was obtained from Dr. Mojallali (Markazi, Iran).

### Preparation of SF

Dried *Bombyx Mori* silk cocoons were cut into small pieces and degummed in boiling aqueous solution of 0.02 M sodium carbonate for 30 min with stirring. The whole mass was repeatedly washed with distilled water (DW) to remove the glue-like sericin protein and dried overnight at room temperature. SF solution was prepared by dissolving degummed silk in 9.3 M LiBr solution at 60°C for 4 h. The fibroin solution was dialyzed in a cellulose membrane based dialysis tube (molecular cutoff 8000) against DW for 3 days, changing the water every 6 h, to remove LiBr. After dialysis, SF solution was centrifuged at 4 °C and 9000 rpm for 20 min. The dialyzed solution was stored at 4 °C for further study.^24^

### Preparation of SFNPs

The applied method for nanoparticles generation in this study is nano-precipitation. The SF was dissolved in DW and the drug was added to this aqueous solution at 1:1, 1:3 and 1:10 weight ratios of drug to polymer. The obtained solutions were stirred for 120 s, and one ml of each ratios was added dropwise to 10 mL acetone under magnetic stirring and stirred for 20 min. Due to the low solubility of SF in acetone and high solubility in water, the drug-polymer mixture precipitated spontaneously as nanoparticles and water diffused in acetone. The produced nanoparticles were separated from dispersion medium by ultracentrifuge (70Ti, Beck man Co., USA) at 20000 rpm and 4°C for 20 min. The nanoparticles were freeze-dried for further studies (by Vaco10-II-E, Zirbus Co., Germany).

### Morphology of nanoparticles

The morphology and structure of obtained nanoparticles at optimal conditions were determined by transmission electron microscope (TEM) and scaning electron microscope (SEM). To obtain TEM images, the nanoparticle solution was placed on a carbon-coated copper grid and then was dried at room temperature. The TEM images were obtained using a Zeiss-EM10C TEM (-80 KV, Germany). The Hitachi S-4160 field-emission scanning electron microscope (FESEM) was used to obtain SEM images.

### Cryopreservation

To prevent the aggregation of SFNPs, cryopreservative agents can be used. For this purpose and before freeze-drying, the prepared nanoparticles (80 mg) were dispersed in 300 µl of different cryopreservative solutions (mannitol, sorbitol, polyethylene glycol [PEG] 4000, and lactose in DW at a concentration of 10% w/v for each of them), and the suspensions were freeze-dried.

### Particle size and zeta potential

The size and zeta potential of nanoparticles were measured by Zetasizer (Nano-ZS, Malvern, UK) equipped with a 633 nm He-Ne laser beam and the scattering angle of 173°. All particle sizes were measured using dynamic light scattering (DLS). The zeta potential of nanoparticles was measured by the Laser Doppler Electrophoresis technique. To evaluate the effect of cryopreservative on the size and polydispersity index (PDI) of nanoparticles, freeze-dried nanoparticles were re-suspended in 1 ml DW, and these characteristics were measured.

#### Fourier-transform infrared spectroscopy

The interaction of the polymer with the drug was studied by Fourier-Transform Infrared Spectroscopy(FT-IR) (Irprestige-21, Shimadzu Co., Japan) spectroscopy using potassium bromide tabs. The range of spectra was in 400-4000 cm^-1^ with an accuracy of 4 cm^-1^.

#### X-ray diffraction

The crystal structure of nanoparticles was characterized by X-ray diffraction (XRD). XRD analysis was performed by INEL, EQUINOX 3000 (France) X-ray diffractometer using 30 kW Cu Kα (λ = 1.5409 A) radiation, in the 2θ range of 0-120°.

### Loading and In vitro release studies

The amount of entrapped 5-FU in nanoparticles was determined by UV-Vis spectroscopy (Shimadzu UV-2450). In order to load 5-FU in nanoparticles, different amounts of drug were added to constant amount of SF solution. Drug-loaded nanoparticles were centrifuged at 20 000 rpm for 30 min at 4°C. After precipitation of the drug loaded nanoparticles, the supernatant was collected and absorption was measured at 254 nm.

The amount of unloaded drug was estimated using UV-Vis spectroscopy, and the loading was calculated by subtracting the amount of drug in supernatant from the initial amount of drug. The entrapment efficiency of the drug in nanoparticles was calculated using following equations:

(1) $$\text{Encapsulation efficiency }\left(\text{EE\%}\right)=\;\frac{\text{the weight of entrapped drug}}{\text{the weight of feeding }}\times 100$$

(2) $$\text{Loading content}=\;\frac{\text{the weight of entrapped drug}}{\text{the weight of drug loaded nanoparticles}}\;\times 100$$

To carry out an *in vitro* release study of the drug from nanoparticles, freeze dried 5-FU loaded nanoparticles were suspended in DW, and the solution was stored in a dialysis membrane (cut-off 12000 Da). The membrane was placed in the under stirring PBS release medium (Phosphate buffer solution at pH 7.4) at 120 rpm and 37 ± 2°C for 144 h. At time intervals, certain amounts of release medium were withdrawn, and absorption was measured using the UV spectrophotometer, and then replenished with fresh PBS (kept at the same temperature). The drug concentration was determined using UV-Vis spectroscopy, with same conditions to loading study. To assure the accuracy of results, all release experiments were repeated three times. The release kinetic was determined by mathematical models; the used models included zero-order, first order, Higuchi, Korsmeyer-Peppas and Hixson-Crowell model.^25^

### Cell viability assay

The cytotoxicity of drug loaded SFNPs, pure drug, and blank SFNPs on MCF7 and HT29 was investigated using Tetrazolium (MTT) assay. These two cell lines were purchased from the Pasteur Institute of Iran. The HT29 and MCF7 cell lines were fed into 96-well tissue culture plates with the density of 5 × 10^4^ cells/mL. After 24 h, 20 µL of each sample was added to each well and plate was incubated in an incubator containing 95% air and 5% carbon dioxide. To evaluate sensitivity of cells to cytotoxic compounds, doxorubicin hydrochloride at concentration of 20 µg/µL was used as a positive control. 72 h post incubation, the culture medium was removed from each well, and 180 μL of fresh medium was replaced. 20 μL of MTT solution was added to wells and plate was incubated for 3 h at 37°C. During this period, living cells produced blue insoluble formazan from the yellow soluble MTT. The reaction was stopped by removing medium and washing wells by PBS. DMSO was added to each well (150 µL/wells) to dissolve formazan crystals, and the solution absorbance was detected with an ELISA plate reader (Hybrid Synergy, Biotech, USA) with a test wavelength of 540 nm^26^ and a reference wavelength of 630 nm to obtain sample signal (OD540-OD630).

The absorbance of the formazan treated wells in the visible region correlates with the number of viable cells as follows:

Viable cells (%) = (T/C) × 100 (3)

Where *C* is the absorbance of control and *T* is the absorbance of treated samples.

## Results and Discussion

### Characterization of nanoparticles

#### The size and zeta potential of nanoparticles

The size, size distribution, and zeta potential of prepared nanoparticles were evaluated that the results of formulations with different ratios of 5-FU to SF (1:1, 1:3, and 1:10) are shown in Table 1 (fresh samples were used for this part of the study). According to Table 1, among these three formulations, the formulation at a ratio of 1:10 )5-FU: SF) had the largest and the most polydisperse nanoparticles. The particle size and the PDI were decreased by increasing amount of 5-FU in the formulations.

**Table 1:** The size, PDI=polydispersity index, and zeta potential of 5-FU loaded silk fibroin nanoparticles with a different drug to polymer ratios

** Weight ratio of 5-FU:SF**	** Size (nm)**	** PDI **	**Zeta potential**
1:1	221.033 ± 2.73	0.093 ± .028	-32.1
1:3	235.767 ± 0.461	0.096 ± 0.03	-32.3
1:10	442.433 ± 2.154	0.297 ± 0.012	-43.6

Size reduction by increasing the drug to polymer ratio can be attributed to the possible conformational change in the SF structure and increasing hydrophobicity of system induced by the drug that is discussed with more details at XRD and FT-IR sections. As shown in Table 1, the zeta potential of nanoparticles was ranged between -32.1 to -43.6 and reduced by increasing the drug to polymer ratio which indicates stability of nanoparticles. Previous studies showed that the nanoparticles are stable at zeta potentials higher than ± 25 mV.^27^

#### FT-IR results

**Figure 1:**
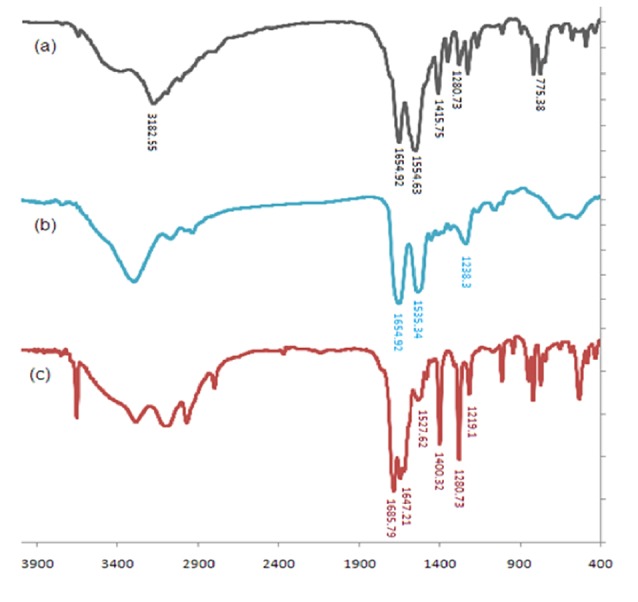
The FTIR of 5-FU(a), silk fibroin (b) and 5-FU loaded silk fibroin nanoparticles (c).

The FT-IR spectra of 5-FU, SF, and 5-FU loaded SFNPs are illustrated in Figure 1. The spectrum of 5-FU (Figure 1a) showed characteristic vibration bands at 775.38, 1280.73, 1415.75, 1554.63, 1654.92, and 3182.55 cm^-1^, which indicate the functional groups of C-H (bend), C-F (stretch), C-N (stretch), N-H (bend), C=O (bend) and N-H (stretch) respectively. The conformational structure of SF can be determined based on the wavenumber location of the absorption bands of amides I. Absorption bands in the range of 1616–1637 cm^−1^ represent enriched β-sheet structures in the silk II form. Absorption bands in the range of 1638–1655 cm^−1^ were attributed to the random coil, 1655–1663 cm^−1^ were related to alpha-helices, and 1663–1695 cm^−1^ were assigned to turns.^24^ According to spectrum of SF (Figure 1b), the vibration band at 1654.92 cm^-1^ demonstrated the presence of amide I (C=O stretching band), and the characteristics vibration bands at 1535.34 and 1238.3 cm^-1^ are attributed to amide II (secondary N–H bending) and amide III (C–N and N–H functionalities), respectively. The position of vibration band of amide I (1654.92 cm^-1^) in the spectrum of SF indicates the random coil of its conformational. In the spectrum of 5-FU loaded nanoparticles (Figure 1c), three observed peaks at 775.38 cm^-1^ (relating to C-H functional group), 1280.73 cm^-1^ (relating to C-F functional group), and 1400.32 cm^-1^ (relating to C-N functional group) confirmed the presence of 5-FU in the nanoparticles, and the peak in the range of 3100-3600 cm^-1^ could confirm the hydrogen bonds between drug and polymer.

#### X-ray diffraction

**Figure 2:**
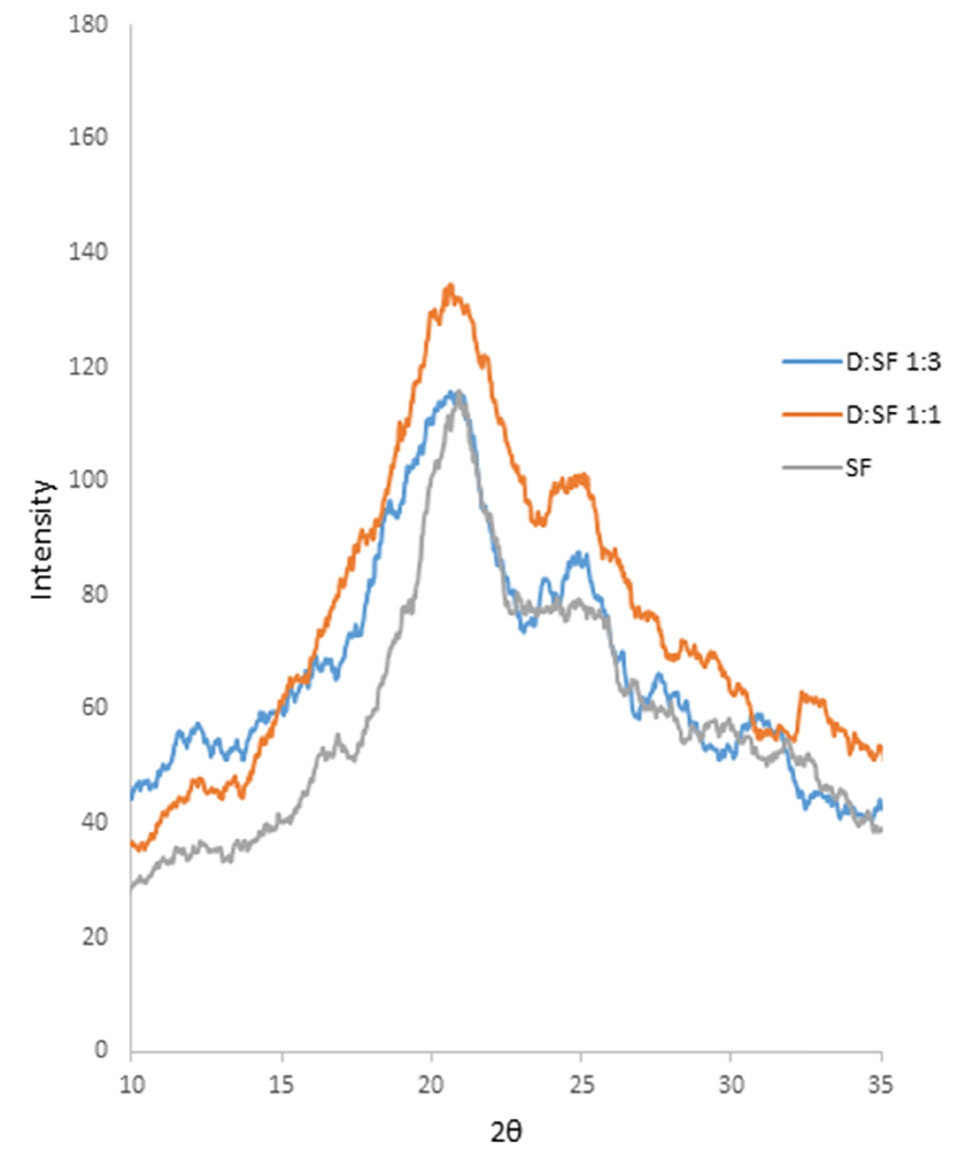
The XRD diffractograms of SF (silk fibroin), D:SF 1:1 (drug to silk fibroin ratio of 1:1), D:SF (drug to silk fibroin ratio of 1:3).

The crystalline structure of materials can be examined by X-ray diffractogram. The diffractograms of SF, 5-FU and drug-loaded nanoparticles at ratios of 1:1 and 1:3 are represented in Figure 2. The diffractogram of SF showed amorphous state and had no crystalline peak which denoted the silk I conformation. In the diffractogram of nanoparticles with 1:1 drug to polymer ratio, the peak at 2*θ* = 20.63° indicates inducing β-sheet crystallization of SF.^10^ The diffractogram of nanoparticles with a drug to polymer ratio of 1:3 was similar to the diffractogram of SF, and it had no significant conformational change to the β-sheet structure. Overly, it could be concluded that formulation with the drug to polymer ratio of 1:1 showed crystalline form, and conformational change to the β-sheet structure was observed for this formulation, which could be attributed to the new inter-molecular hydrogen bonds between drug and SF.^28^ This could be one of the possible reasons for the smaller size of nanoparticles obtained at 1:1 drug to polymer ratio. It has been approved that promoting β-sheet crystallization reduces the hydrodynamic radius of the protein chains and increases intra-molecular hydrogen bonding between them.^29,30^ For ratio of 1:1, the conformational change from random coil to β-sheet structure resulted in shrinking of nanoparticles and reduction in their size.

#### Effect ofcryopreservativeson the size and PDI of nanoparticles

**Figure 3:**
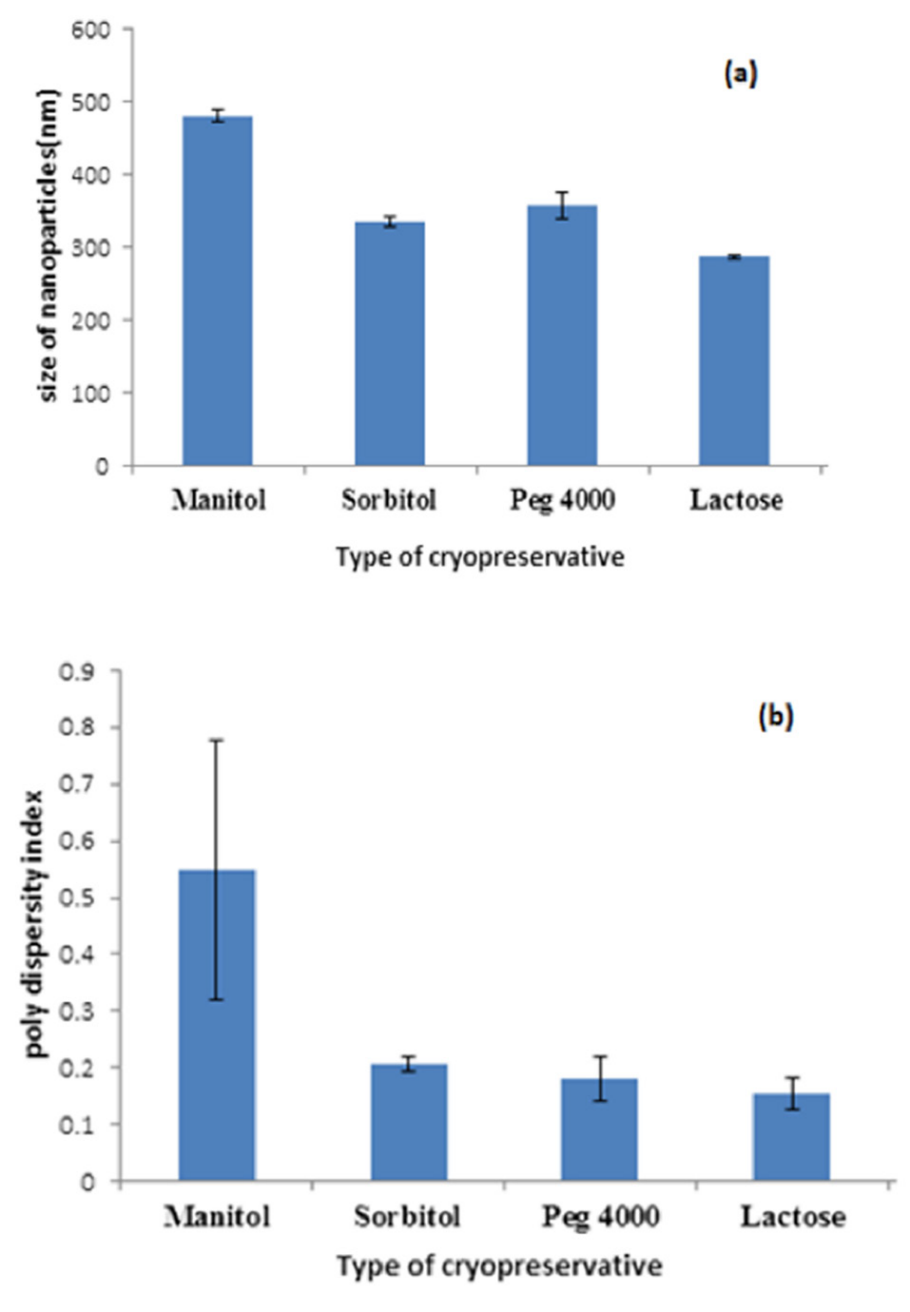
The size (a) and the PDI = polydispersity index (b) of silk fibroin nanoparticles in the presence of different cryopreservatives

**Figure 4:**
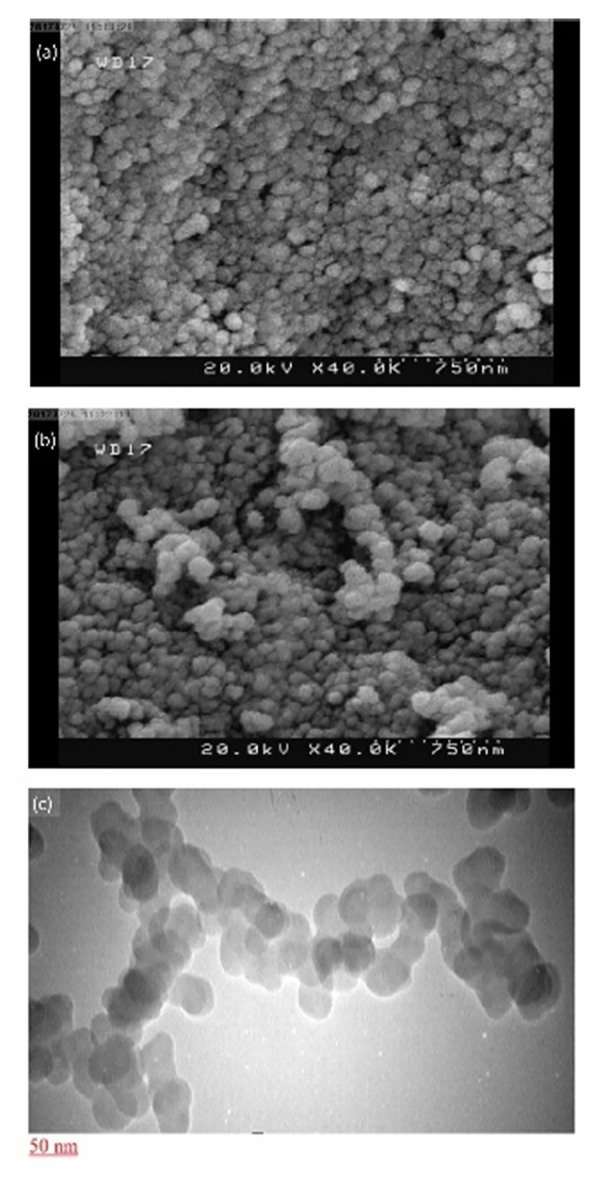
SEM image of 5-FU loaded silk fibroin nanoparticles at ratio 1:1 without cryopreservative (a) and with cryopreservative (b), TEM image of 5-Fu loaded silk fibroin nanoparticles (c).

According to the literature review, SF nanoparticles suffer from the drawback of aggregation during freeze-drying which hinders the effective dispersion and its application as a dosage form.^31^ It is worthy to mention that adding the cryopreservatives is beneficial due to avoiding the aggregation during freeze-drying. Thus, release profiles severely affected by the type of performed cryopreservative and it is necessary to choose a proper cryopreservative. To examine the effect of cryopreservatives on the size and PDI of nanoparticles, different types of cryopreservatives were utilized, and the obtained particles were compared regarding to the size and size distribution. The size and PDI of freeze-drying nanoparticles at a ratio of 1:1 in presence and absence of cryopreservatives are shown in Figure 3. A glance at this figure, apparently indicates that among the four performed cryopreservatives, the nanoparticles in the presence of 10 w/v% aqueous solution of lactose were the smallest and the most homogenous nanoparticles with a narrowest standard deviation of size and PDI (the size of 286.7 nm and a PDI index of 0.154). The TEM and SEM micrographs of freeze-dried nanoparticles with cryopreservative also confirms these results (Figure 4b & c). This result reflected that SF nanoparticles could be well dispersed in release medium in the presence of lactose, and 10% lactose was chosen as the most suitable cryopreservative.

#### TEM and SEM results

The SEM photograph of 5-FU loaded SFNPs at the ratio of 1:1 without any cryopreservative (Figure 4a) showed that they are spherical but particles are aggregated. Also, it should be noted that the particle size in SEM is smaller than one measured by DLS. Similar result was observed by other researchers for other nano-hydrogels. Hydrogels are swellable, and their volume can increase several times of magnitudes in aqueous environments comparing to dry state.^32^ To evaluate effect of cryopreservative on morphology of nanoparticle, TEM micrograph of SFNPs at ratio of 1:1 and at present of 10% lactose was prepared. A glance to Figure 4b & c indicates spherical nanoparticles, and borders of particles are not fused together.

#### Loading results

**Figure 5:**
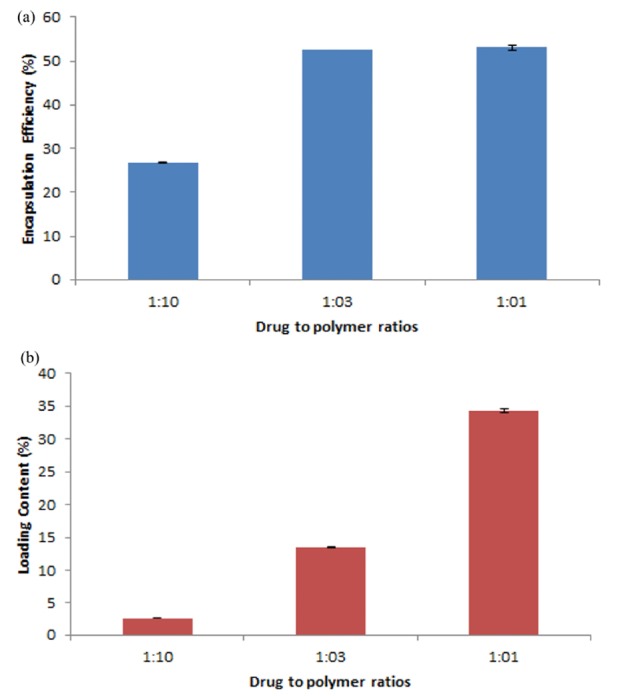
Loading efficiency (a) and loading content (b) of 5-Fu loaded silk fibroin nanoparticles.

The amount of 5-FU encapsulated inside the SF nanoparticles was measured using UV-Vis spectroscopy in triplicate. The amount of polymer was kept constant while the amount of drug was varied to obtain the 1:1, 1:3, and 1:10 ratio of drug to the polymer. The loading efficiency and loading content of drug at these three ratios of drug to the polymer are demonstrated in Figure 5a & b. The loading efficiency was found 52.32 ± 0.62, 51.84 ± 0.67, and 26.6 ± 0.22% for formulations with 1:1, 1:3, and 1:10 drug to polymer ratios, respectively. The highest loading efficiency was achieved at the ratio of 1:1. The drug to polymer ratio of 1:1 with the loading content of 34.34 ± 0.27, had also the highest loading content. According to XRD analysis, for the formulation of the drug to polymer ratio of 1:1, the conformational transition from random coil structure to β-sheet was observed which can be atributed to inter-molecular hydrogen bond between 5-FU ans SF. The presence of β-sheet structure results in reduced diffusion and accumulation of 5-FU in the nano-hydrogel of SF. Increasing the β-sheet content in the SF causes increasing the drug loading in the nanoparticles. According to low loading efficiency and content in ratio of 1:10, we did not evaluate it for other characterizations.

#### The in vitro releasestudy

**Figure 6:**
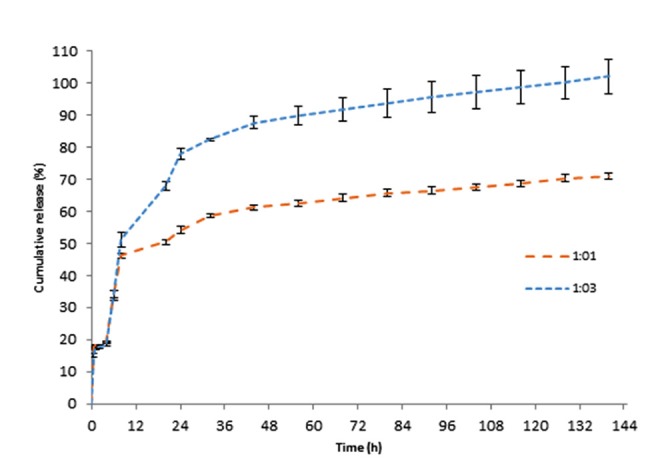
The release profile of 5-FU from 5-Fu loaded silk fibroin nanoparticles (at ratios 1:1 and 1:3).

The release profile of 5-FU from two formulations with 1:1 and 1:3 ratios of drug to polymer is demonstrated in Figure 6a & 6b. The results revealed that release of 5-FU from nanoparticles in 1:1 ratio of drug to polymer was slower than 1:3 ratio. In the formulation with ratio of 1:3, up to 70% of the drug was released in 20 h, while the formulation with ratio of 1:1 released 71% of the drug in sex days. According to aforementioned XRD results, the formulation with 5-FU to SF ratio of 1:1 consists of the crystalline structure of SF while the formulation with 5-FU to SF ratio of 1:3 was in an amorphous state. Therefore, the slower release of the formulation with ratio of 1:1 could be attributed to its crystalline structure, while in the amorphous state the drug release occurs faster. It seems that intercellular hydrogen bonds and van der Waals forces between 5-FU and SF after induction of β-sheet is enough strong to prevent release a part of 5-FU. Probably, this part of 5-FU cannot be release until degradation of SFNPs. The release kinetic of this drug delivery system was evaluated that after the match of the data with different models, the Korsmeyer-Peppas was the most appropriate model (R^2^ = 0.9876).

#### In vitro cytotoxicity

**Figure 7:**
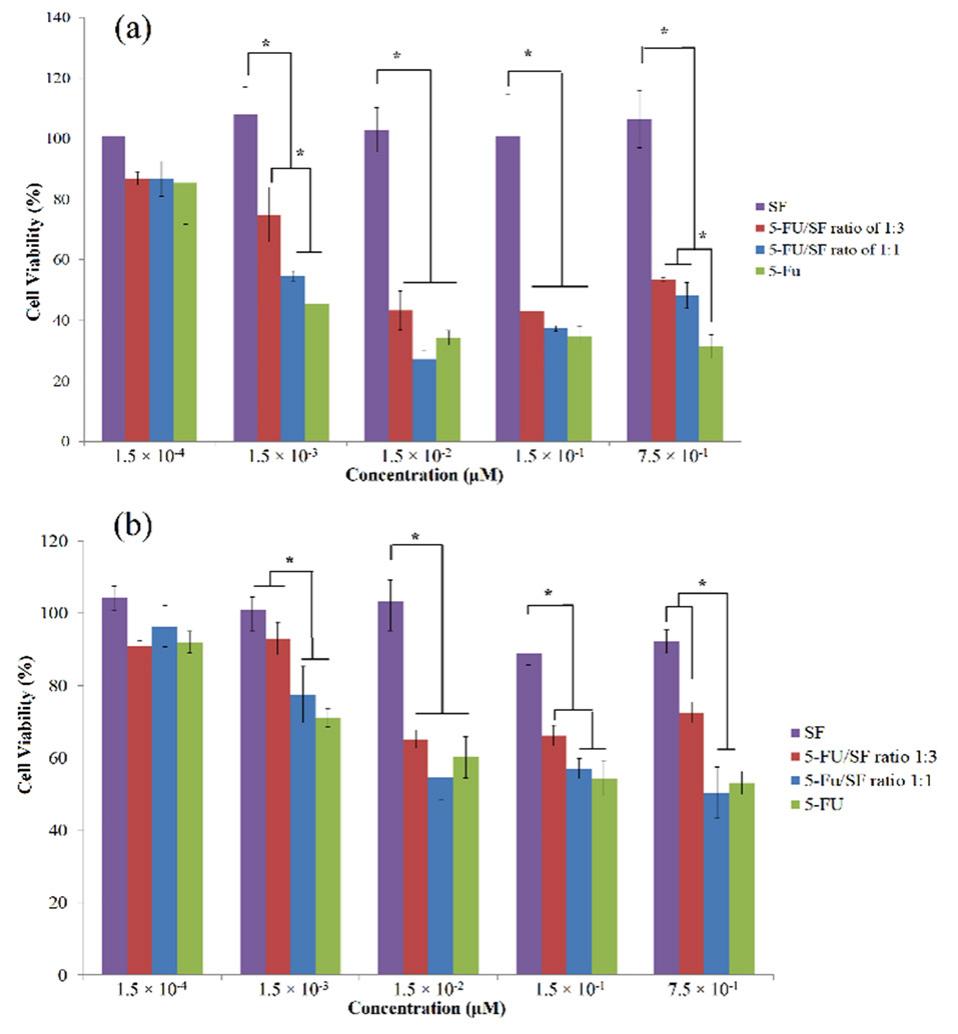
Cytotoxic activity of 5-Fu loaded silk fibroin nanoparticles (at ratios 1:1 and 1:3), 5-Fu and SF (silk fibroin) on MCF7 (a) and HT29 (b) cell lines. The relative cell viability read for the control (tissue culture polystyrene from culture plates) after 72 h of incubation was taken as the reference (100%). 40 µg of doxorubicin hydrochloride was used as a positive control. Data are expressed as the mean ± SEM of three separate experiments. Significance was calculated by ANOVA (*P ≤ 0.05).

The viability of HT29 and MCF7 cells in the presence of 5-FU loaded nanoparticles (at ratios of 1:1 and 1:3), SF and the free drug were investigated to evaluate the cytotoxic effects of drug loaded nanoparticles on these cancerous cell lines. The cytotoxic activity of samples on HT29 and MCF7 cell lines is illustrated in Figure 7. Pure SF showed no cytotoxic activity on both cell lines, and drug-loaded nanoparticles showed efficient cytotoxicity at ratio of 1:3 and 1:1 while free 5-FU had similar potency to ratio of 1:1; these results are in agreement to release study. Drug release is sustained in NPs, and a part of drug cannot be release even after 144 h. SF degradation is slow and its degradation inside a cell or physiological condition is not fast. In fact, accessible drug in NPs is less than free drug. Kundu et al have shown that SFNPs can be efficiently uptaken by cells. Therefore, the uptake cannot be a reason for lower activity of drug loaded SFNPs in compare to free 5-FU.^33^ Although further study is needed in this case.

According to Figure 7, HT 29 cells were more resistance to the 5-FU and drug loaded NPs, and neither free drug nor encapsulated one could not reach to IC_50_ in this cell line. MCF7 was more sensitive, and IC_50_ value of free drug and NPs at the ratio of 1:3 was around 0.01 mg/mL while this value was less than 0.01 mg/mL for the ratio of 1:1. The higher efficiency of NPs at ratio 1:1 is attributed to the higher drug content of this formulation, which caused a higher amount of drug in the cells in compare to nanoparticles at ratio of 1:3.

## Conclusion

This study was planned to achieve a promising controlled release drug delivery system for carrying 5-FU, as a potent anticancer drug. SFNPs were found proper candidates for delivery of a hydrophilic drug such as 5-FU. The size and PDI of freshly prepared nanoparticles were found desirable with the minimum value of 221.03 ± 2.73 nm and 0.093± 0.028, and these values were 286.7 nm and 0.154 for freeze dried sample using lactose as the appropriate cryopreservative. At the highest ratio of drug to polymer (5-FU to SF ratio of 1:1), the obtained nanoparticles possessed crystalline portions due to a conformational change in the structure of SF and induction of β-sheets. This caused increasing the loading efficiency, and up to 52.32 ± 0.62% loading was achieved for the ratio of 1:1. The high loading efficiency, the sustained release pattern, and the high cytotoxicity of the obtained nanoparticles at ratio of 1:1 on MCF7 were some of the advantages of these designed drug carrier. It could be concluded that these nanoparticles could be successfully performed as the new and promising delivery systems for the cancer treatment with high loading capacity and sustained release profile.

## Ethical Issues

Not applicable

## Conflict of Interest

The authors report no declarations of interest.

## Acknowledgments

The authors gratefully acknowledge the Research Council of Kermanshah University of Medical Sciences (Grant Number: 93368) for the financial support. This work was performed in partial fulfillment of the requirements for Pharm.D of Hamid Rahmani, in Faculty of Pharmacy, Kermanshah University of Medical Sciences, Kermanshah, Iran.
